# Repeatability of lipid layer thickness using LipiView® following removal of contact lenses and its relationship to comfort

**DOI:** 10.1111/opo.13445

**Published:** 2025-01-18

**Authors:** Mukesh Kumar, Simin Masoudi, Ajay Kumar Vijay, Thomas John Naduvilath, Srikanth Dumpati, Ankit Raj, Mark Willcox

**Affiliations:** ^1^ School of Optometry and Vision Science University of New South Wales Sydney New South Wales Australia; ^2^ Brien Holden Vision Institute University of New South Wales Sydney New South Wales Australia; ^3^ LV Prasad Eye Institute Hyderabad India

**Keywords:** CLDEQ‐8, contact lens, LipiView, ocular comfort, repeatability

## Abstract

**Purpose:**

To assess the repeatability of lipid layer thickness (LLT) measurement using the LipiView® interferometer after daily disposable contact lens (CL) wear and correlation with ocular comfort in soft contact lens wearers.

**Methods:**

A prospective study was conducted over two consecutive months, wherein CL wearers (*n* = 20) wore either Somofilcon A or Verofilcon A daily disposable CLs in a crossover design, switching lenses after 1 month. The pre‐corneal tear film LLT was measured at the end of each month after CLs had been worn for at least 6 h. Using the LipiView® interferometer, three measurements of the LLT (average, maximum and minimum) were recorded. Subjects' symptoms were evaluated with the Contact Lens Dry Eye Questionnaire‐8 (CLDEQ‐8) and correlations sought between post‐lens pre‐corneal tear film LLT and symptoms.

**Results:**

The average, maximum and minimum intraclass correlation coefficients (ICC) of LLT measurements at baseline were 0.57, 0.39 and 0.66, respectively, indicating poor (ICC < 0.4) to moderate (≥0.4, <0.75) repeatability. Coefficients of repeatability (CR) were 20.4, 24.8 and 20.8, respectively. After daily disposable CL wear, the ICC values were 0.66, 0.72 and 0.63 (indicating moderate repeatability), with CR values of 25.7, 32.0 and 23.3, respectively. Although all the ICC values of the pre‐corneal LLT increased after daily disposable lens wear indicating improved repeatability, the CR values also increased, indicating larger spread of data. However, in both cases, these increases were not significantly different from baseline. There were no significant differences in pre‐corneal LLT between the two lens types and no significant correlation with comfort scores (baseline: *r* = −0.11, *p* = 0.67; Verofilcon A lenses: *r* = 0.19, *p* = 0.45; Somofilcon A lenses *r* = 0.13, *p* = 0.62) for either lens.

**Conclusions:**

The repeatability of average, maximum and minimum LLT measurements performed by LipiView remained stable during CL wear. There was no significant correlation between LLT and comfort scores.

**Key points**

- The repeatability of the LipiView interferometer remains stable after contact lens wear.
- Lipid layer thickness was not associated with contact lens comfort for daily disposable silicone hydrogel lenses.
- No significant correlations were found between Contact Lens Dry Eye Questionnaire scores and lipid layer thickness.

## INTRODUCTION

Over 140 million individuals worldwide use contact lenses (CLs) for various purposes, including vision correction, sports and cosmetic reasons.^1^ Interestingly, the literature suggests that the number of people who stop using CLs each year is approximately equal to the number of new users adopting them.^2^, ^3^

The tear film is essential for maintaining ocular health and comfort, and disturbances to its stability can result in dryness and discomfort.^4^ The lipid layer, the outermost layer of the preocular tear film, plays a vital role in tear film function and stability. CL wearers experience various biophysical changes in their tear film.^5^ For example, the tear film lipid layer is generally thinner in soft CL wearers than non‐CL wearers,^5^ and this is related to increased evaporation of tears during CL wear (which approximately doubles) and is possibly related to the increased rapidity of tear thinning during CL wear.^5^ It has been hypothesised that the thickness of this lipid layer and pre‐lens tear film thinning are closely linked to the likelihood of CL discontinuation.^6^, ^7^ Therefore, assessment of the tear lipid layer may provide an indication of ocular comfort during CL wear. Each type of CL material has specific characteristics that affect the dynamics of the tear film.^8^ For example, silicone hydrogel lenses are noted for their excellent oxygen permeability, but they can also cause lipid deposits, which might alter the structure of the tear film.^7^, ^9^

Various devices are available for measuring the thickness of the lipid layer, most of which utilise optical interferometry. These devices include the Tearscope plus (keeler.co.uk), EasyTear Viewplus (easytear.it), bon Polaris (bonOptic.com), Keratograph 5M (K5M; Oculus.co.usa), LipiView® (jnjvisionpro.com/en‐us/products/lipiview‐ii‐ocular‐surface/) and the Ocular Surface Analyzer (sbmsistemi.com/en/).^10^, ^11^, ^12^, ^13^ All these devices share a common underlying technology, where the measurement is performed non‐invasively by observing interference fringes. These fringes enable the lipid layer's thickness to be calculated.

Tearscope Plus, EasyTear Viewplus, OSA and bon Polaris provide subjective and qualitative data. The observer compares the image they see with an existing classification of lipid layer thickness, which is divided into five different categories, as previously described by Gullion.^14^ However, LipiView® offers software that quantitatively measures the thickness of the lipid layer. This device has found application in various scenarios, including assessment of the lipid layer thickness (LLT) in dry eye diseases,^15^ dry eye treatment^16^ following the use of glaucoma medications^17^ and after cataract surgery.^18^

Clinical measurements need to have good reliability and repeatability to aid with the delivery of high‐quality clinical service. Repeatability refers to how closely scores or ratings obtained under similar conditions align with each other; reliability of data is an assessment of whether the instrument gives the same results each time it is used (under defined settings and clinical groups).^19^, ^20^ Previous studies have validated the repeatability of LipiView® assessment in both healthy^21^ and dry eyes.^22^ However, there are no studies that have examined the repeatability specifically in the context of managing CL patients. Considering the lipid layer plays a crucial role in CL wear, significant alterations during lens wear could notably affect eye comfort. Therefore, it is necessary to establish whether an instrument can precisely differentiate subtle variations, essentially evaluating the sensitivity of the measurements.

Since the lipid layer contributes to tear film stability,^23^, ^24^ it is important to understand the reliability of measurements of the lipid layer in CL wearers. Measurements of the lipid layer may help refine clinical assessments for CL wearers. By examining repeatability in this context, this study aimed to provide more consistent and accurate data regarding the effects of CLs on the tear film.

Although prior research has examined LLT in individuals not wearing CLs,^19^ the present study specifically investigates the repeatability of these measurements in CL wearers. This focus is important because gaining insights into LLT repeatability within this group can enhance our understanding of the ocular surface dynamics related to CL usage. A consistent decrease in LLT across measurements might indicate that the CL material is disrupting the lipid layer, leading to increased tear evaporation and more significant discomfort for the wearer.

This study aims (1) to evaluate the repeatability of LLT measurements using the LipiView® interferometer and (2) to assess any correlation between LLT and Contact Lens Dry Eye Questionnaire‐8 (CLDEQ‐8) scores with the use of two different types of daily disposable silicone hydrogel (Si‐Hy) CLs in healthy subjects.

## MATERIALS AND METHODS

This study received ethical approval from the University of New South Wales Human Research Ethics Advisory Panel (HC220806) and adhered to the tenets of the Declaration of Helsinki. Inclusion criteria were defined as participants aged between 18 and 40 years, current CL wearers who used CL at least 5 days a week for 6 h per day. Participants with active ocular surface disease such as active corneal infection, allergy, corneal oedema and iritis were excluded as were those using eye drops. Individuals with neurological disorders such as epilepsy, taking systematic medication known to affect tear film dynamics or who were pregnant or lactating were also excluded from participating in the study.

### Study design

**FIGURE 1:**
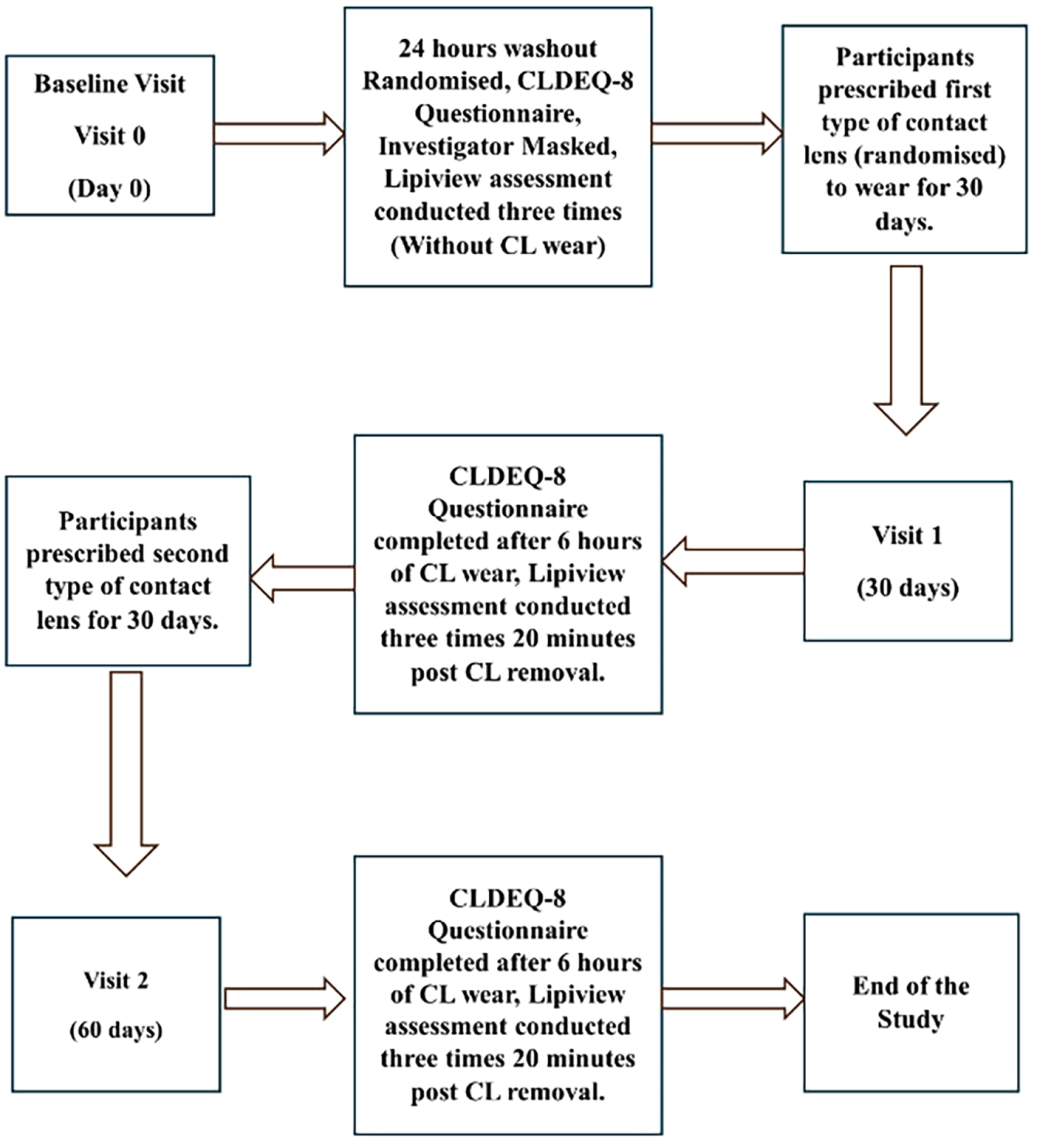
Flow chart of the study visits. CL, contact lens; CLDEQ‐8, Contact Lens Dry Eye Questionnaire‐8.^25^

The study was conducted as a prospective, randomised, bilateral, crossover trial. The investigator was masked to the types of the lenses being used by participants. The research was carried out for 2 months. CL wearers were asked to cease lens wear for 24 h and then wore one of two commercial daily disposable soft Si‐Hy CLs—Somofilcon A (Clariti 1 day, CooperVision, coopervision.com/contact‐lenses/clariti‐1‐day) or Verofilcon A (Precision 1, Alcon, precision.myalcon.com/au) in both eyes in a crossover design. Symptoms with each lens were documented using the validated CLDEQ‐8,^25^ which has been demonstrated to possess high sensitivity and specificity for diagnosing dry eye associated with CL wear. The LLT of each CL wearing eye was assessed for each lens type using the LipiView®. Participants were advised not to rub their eyes during the measurements, blink forcefully or blink excessively, and compliance was monitored during testing. Blink rates were observed and kept consistent by encouraging natural blinking throughout the process. Participants were instructed to avoid screen use or reading for at least 30 min before testing. Measurements were taken three times at each visit (Figure 1): the first visit after 24 h of not wearing their habitual lenses, the second visit after 30 days of daily disposable wear of one lens type and the last visit after 30 days of daily disposable wear of the second lens type. Measurements at the second and last visit were taken 20 min after the removal of CLs.

### Lipid layer thickness measurement

The LipiView® Ocular Surface Interferometer (Tear Science Inc, jnjvisionpro.com/en‐us/products/lipiview‐ii‐ocular‐surface/) was used to measure the LLT. To obtain the measurements, the instrument was aligned with the lower third of the cornea, positioned approximately 1 mm above the inferior tear meniscus and manually focused.^22^ Throughout the study, the same investigator operated the LipiView® in a room with controlled temperature (24°C ± 1°C). For each study participant, the following measures were recorded: the average tear film thickness obtained from all frame averages, as well as the maximum and minimum thickness. LLT measurements were obtained for only one eye, randomly assigned, of each participant. It is worth noting that the LipiView® has a maximum limit of 100 interferometric colour units (ICU). Any readings reaching 100 ICU were not considered valid and excluded from the study.^19^

### Data analysis

A sample size calculation was performed from LLT data obtained in a previous study that compared measurements between groups of CL wearers with low (7.67 ± 2.94) and high (16.38 ± 4.16) CLDEQ‐8 scores.^26^ With a baseline mean value of 2.14 (SD = 1.33) and aiming to detect a clinically significant post‐intervention difference of 0.61 (SD = 0.80), the required sample size was determined. The calculation was conducted with a type I error rate (α) of 0.05 and a statistical power (1 − β) of 80%. The sample size required to achieve these parameters was 10 participants. With a possible 20% dropout rate, a minimum of 12 people were required to be enrolled in the study. All data were analysed using SPSS software version 26.0 (ibm.com). Normality was tested using the Kolmogorov–Smirnov test. The repeatability across three measurements was assessed by repeated‐measures analysis of variance. If there was no significant difference between the repeated measurements, within‐subject standard deviation (Sw) and coefficient of repeatability (CR) were used to summarise the extent of repeatability. Concordance between repeats was also summarised using the intraclass correlation coefficient (ICC). Correlation of the average of three repeats with within‐subject standard deviation was assessed using scatter graphs and Pearson's correlation. Correlation of the average LLT with CLDEQ‐8 total scores was also analysed using scatter graphs and Pearson's correlation. The level of significance was set at 5%.

## RESULTS

Informed consent was obtained from 20 participants who were all experienced healthy CL wearers between 18 and 37 years of age (mean 25.4 ± 6.1 years) and included 16 females. At the baseline visit, the pre‐corneal LLT ranged from 20 to 100 nm and the average, maximum and minimum measurements from the LipiView® were 54.7 ± 14.7 (95% confidence interval [CI] 50.1–59.2), 72.9 ± 17.4 (95% CI 67.6–78.3) and 39.8 ± 15.5 (95% CI 35.6–43.9), respectively.

After 30 days of wearing Somofilcon A lenses, the average, maximum and minimum LLT measurements from LipiView® were 53.3 ± 15.9 (95% CI 45.1–61.5), 71.7 ± 16.9 (95% CI 62.9–80.4) and 36.7 ± 10.7 (95% CI 31.1–42.2), respectively. Similarly, following 30 days of Verofilcon A lens wear, the respective measurements were 52.7 ± 14.5 (95% CI 45.3–60.3), 70.8 ± 20.1 (95% CI 60.4–81.1) and 39.5 ± 14.3 (95% CI 32.1–46.9).

As shown in Table 1, at the baseline visit, the pre‐corneal LLT across three repeated readings ranged from 51.7 to 53.5, 67.0–71.9 and 34.9–37.9 nm for the mean, maximum and minimum thicknesses, respectively. There was no significant difference between the three repeated readings (repeated measures ANOVA *p* > 0.47). Similarly, after 30 days of lens wear, there was no significant difference between the three repeated readings of average, maximum and minimum LipiView® measurements with Somofilcon A (repeated measures ANOVA *p* > 0.85) or with Verofilcon A (repeated measures ANOVA *p* > 0.12). When both lenses were analysed together, there was no significant difference between the three repeated readings of average, maximum and minimum LipiView® measurements (repeated measures ANOVA *p* > 0.35). The interaction of lens type (Somofilcon A, Verofilcon A) with repeats was also not significant (*p* > 0.76). Therefore, the subsequent within subject standard deviation was derived for the combination of lens types.

**TABLE 1:** Repeatability of Lipiview parameters measured after using contact lenses.

Measurement	Visits	Reading 1	Reading 2	Reading 3	RM ANOVA, *p*‐value	Within SD (Sw)	CR	ICC
Average LLT (nm)	Baseline	51.9 ± 14.6	53.5 ± 13.1	51.7 ± 10.2	0.79	10.2	20.4	0.57
	After lens wear	52.9 ± 18.9	53.6 ± 18.4	53.4 ± 14.8	0.96	12.8	25.7	0.66
Maximum LLT (nm)	Baseline	71.0 ± 18.5	71.9 ± 15.2	67.0 ± 14.7	0.50	12.4	24.8	0.39
	After lens wear	75.5 ± 21.7	72.9 ± 21.2	72.5 ± 18.5	0.47	16.0	32.0	0.72
Minimum LLT (nm)	Baseline	34.9 ± 11.6	37.9 ± 13.6	37.4 ± 12.0	0.47	10.4	20.8	0.66
	After lens wear	37.3 ± 14.6	40.2 ± 16.9	40.0 ± 12.4	0.34	11.7	23.3	0.63

Abbreviations: CR, coefficient of repeatability; ICC, intraclass correlation coefficient; LLT, lipid layer thickness; RM, repeated measures; Sw, within‐subject standard deviation.

The within‐subject standard deviation obtained from the three repeated readings was close to 11 nm without lens wear from the three variables (average, maximum and minimum) and increased slightly to 13 nm units after lens wear. Correspondingly, the coefficient of repeatability ranged from 20.4 to 24.8 nm without lens wear for the three variables and from 23 to 32 nm after lens wear. The ICC for the three readings ranged from 0.39 to 0.72, which indicates poor (<0.4) to moderate (≥0.4 to <0.75) concordance between readings.^27^

**FIGURE 2:**
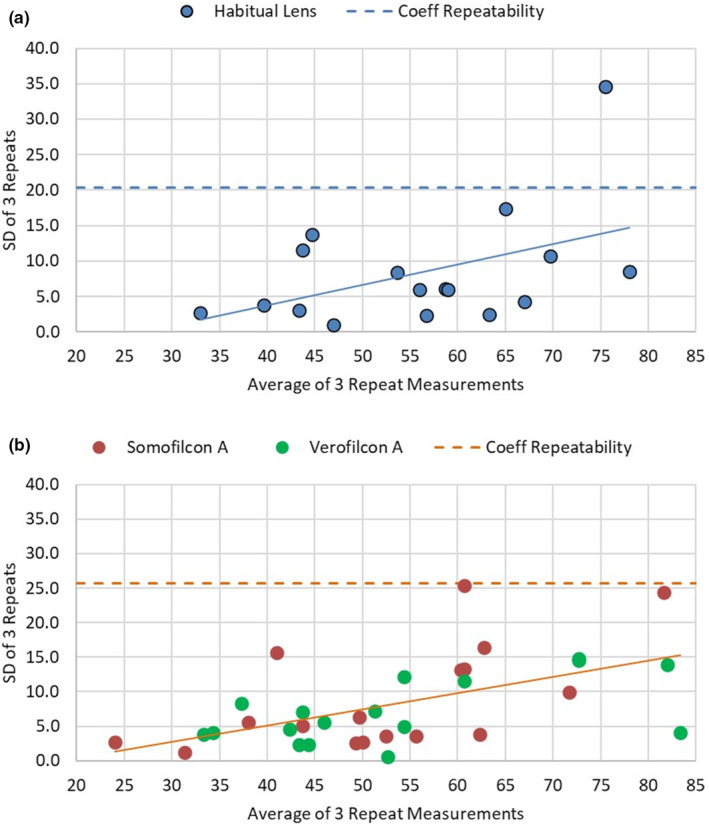
Scatter graph denoting repeatability of lipid measurements with habitual lens (a) and with test lenses (b), where *x*‐axis is the average of three repeat measurements for each participant and *y*‐axis is the corresponding within‐subject standard deviation (SD). The unbroken line is the linear line of best fit and the dashed line represents the coefficient (Coeff) of repeatability for the entire sample.

The scatter graphs (Figure 2) of the average of the three readings and the within‐subject standard deviation indicated a significant correlation for the post lens wear data (*r* = 0.56, *p* = 0.001), but showed only a non‐significant trend before lens wear (*r* = 0.46, *p* = 0.07). These positive correlations indicate that the within‐subject variance increased with higher LLT average values.

**FIGURE 3:**
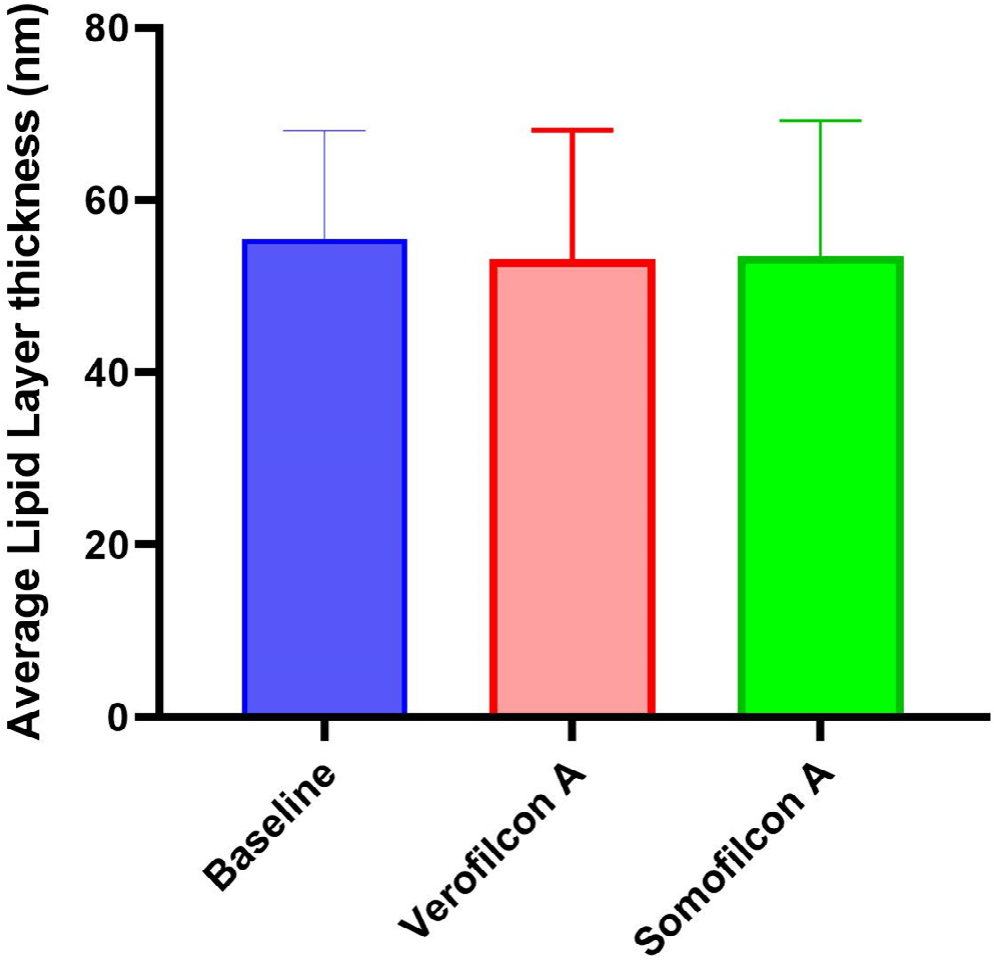
Average values for the lipid layer thickness measured with LipiView at baseline and with different contact lenses. Errors bars represent standard deviations.

When each lens effect (Figure 3) was compared with pre‐lens wear, average, maximum and minimum LLT reduced slightly but not significantly with Somofilcon A (mean difference −2.1 ± 21.9, *p* = 0.69; −2.5 ± 22.5, *p* = 0.64; −6.7 ± 24.6, *p* = 0.27), respectively, and with Verofilcon A lens (mean difference −2.9 ± 14.4, *p* = 0.42; −3.48 ± 25.7, *p* = 0.59; −3.9 ± 21.7, *p* = 0.46), respectively. These differences were not significant, even when average data were log transformed (*p* = 0.30 for Somofilcon A and *p* = 0.61 for Verofilcon A).

**FIGURE 4:**
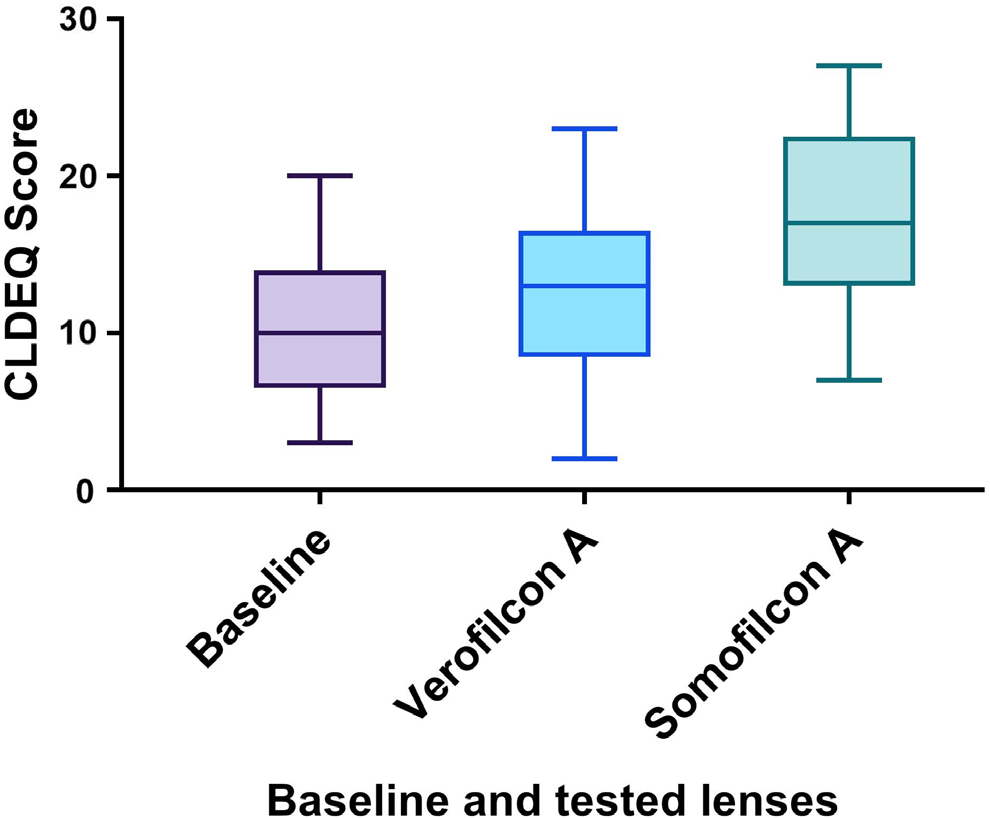
Subjective evaluation at baseline and with different contact lenses using the Contact Lens Dry Eye Questionnaire (CLDEQ) scores.

**FIGURE 5:**
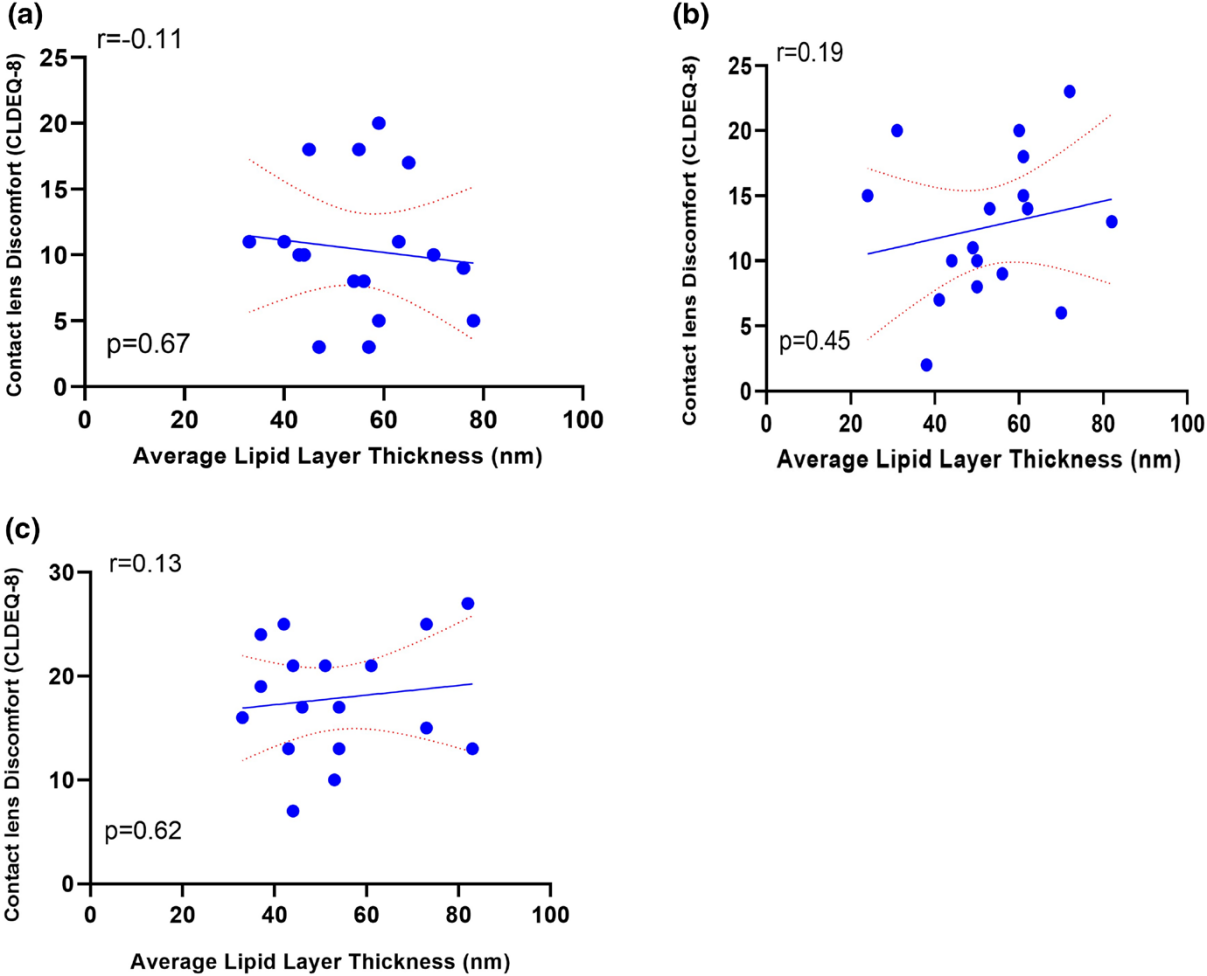
Association between contact lenses dry eye questionnaire (CLDEQ) scores and average lipid layer thickness, (a) Baseline, (b) Verofilcon A lenses, (c) Somofilcon A lenses. CLDEQ‐8, Contact Lens Dry Eye Questionnaire‐8.

The CLDEQ‐8 scores at baseline 24 h post‐lens wear were 10.4 ± 5.2 (95% CI 7.7–13.0). Following 30 days of lens wear, CLDEQ‐8 scores increased to 12.6 ± 5.5 (95% CI 9.7–15.5) with Verofilcon A lenses and 17.9 ± 5.6 (95% CI 14.9–20.8) with Somofilcon lenses (Figure 4), which were significantly different from each other (*p* = 0.009; paired *t*‐test). Statistical analysis revealed no significant correlation between CLDEQ‐8 scores and LLT (Figure 5) at the baseline visit (*r* = −0.11, *p* = 0.67) and after 30 days of lens wear (Verofilcon A: *r* = 0.19, *p* = 0.45, Somofilcon A: *r* = 0.13, *p* = 0.62). These results indicate that the CL discomfort symptoms reported by participants did not correlate with the LLT, regardless of the type of lens worn.

## DISCUSSION

Measuring the LLT is important for helping to diagnose and evaluate dry eye conditions. Utilising non‐invasive or minimally invasive and objective tests offers significant benefits compared to more invasive and subjective methods for assessing the parameters of the ocular surface. Consequently, it is essential to determine how reliably these measurements can be repeated.

In the current study, the pre‐corneal LLT was measured using LipiView® in daily disposable Si‐Hy CL wearing participants. To date, no data exists on the variability of the pre‐corneal LLT over time for CL wearers.

The average baseline LipiView® LLT was 54.7 ± 14.7 nm. This is consistent with mean values reported in previous studies: 56.3 ± 16 nm,^19^ 59.70 ± 17.21 (observer 1),^21^ 56.70 ± 16.47 (observer 2)^21^ and 53.53 ± 14.59^22^ in non‐CL wearers. In contrast, one prior study reported a LipiView® average of 65.0 ± 19.1 nm in healthy controls and 54.2 ± 17.9 nm in individuals with meibomian gland dysfunction.^28^ This discrepancy may be attributed to differences in the age demographics between the studies. A recent investigation concluded that Lipiview® was a useful non‐invasive tool in the evaluation of LLT in patients after refractive surgery, showing that the data ranged from a baseline value (pre‐operative) of 53.38 nm to (post‐operative) 53.32 nm after 6 months (*p* = 0.89).^29^

The current study demonstrated mild to moderate repeatability for average and minimum pre‐corneal LLT and in baseline CL wearers (ICC 0.57 and 0.66, respectively). However, there was poorer repeatability for maximum pre‐corneal LLT (ICC 0.39).^27^ After wearing the study CL, mild to moderate repeatability was observed for average, minimum and maximum pre‐corneal LLT, with ICC of 0.66, 0.72 and 0.63, respectively (Table 1). The average, minimum and maximum LLT measurements demonstrated the lowest coefficient of repeatability (20.4–24.8 nm—see Table 1) at 1 day before CL wear, indicating better between‐visit agreement compared with after CL wear (23.0–32.0 nm). However, in another group consisting of 90% non‐CL wearers,^22^ the coefficient of repeatability of the LipiView® device for inter‐observer repeatability on the same day was 16 nm, while the intra‐observer coefficient of repeatability was 13 nm.^22^ These repeatability values are notably lower than the day‐to‐day coefficients of repeatability observed in the current study. This discrepancy might be attributed to CL wear having a disruptive effect on the pre‐corneal LLT. The lipid layer is known to be influenced by factors such as the palpebral aperture size and blink rate,^30^, ^31^ and blink rate can be affected by CL wear.^32^ This variability of LLT is evident in the differences observed across different days (Table 1). It is further highlighted by the increased standard deviations for each variable on different days. This has important implications for contralateral and longitudinal studies, as it is crucial to establish baseline differences before assessing the effects of various treatment on the eye over time. Prior studies have assessed the accuracy of quantitative pre‐corneal LLT measurements using a new spectral interferometer,^33^, ^34^, ^35^ which is not currently commercially available. That previous investigation reported a high correlation between repeated measures of lipid thickness (Spearman's *r* = 0.84).^35^ However, correlation alone does not serve as a suitable indicator of repeatability, as it measures association rather than agreement.

When assessing CL wearers in the absence of lens wear, Glasson et al.^36^ reported no discernible differences in the LLT between participants who tolerated CLs (based upon the McMonnies dry eye questionnaire) and those who did not, aligning with the findings of the present study. This previous study used a subjective grading scale with slit lamp biomicroscopy to classify the pre‐corneal LLT.

CL wear can significantly alter the tear film, including changes in LLT.^35^ Indeed, Si‐Hy lenses have been associated with a reduction in LLT due to increased evaporation rates and disruption of the tear film structure.^34^, ^35^ Furthermore, CL wear can alter the structure of meibomian glands that deliver the majority of the tear film lipid layer to the tear film.^23^, ^37^

Although LLT can fluctuate during CL wear, assessing it after lens removal provides valuable information about the condition of the ocular surface following lens wear. While not a true baseline, this post‐wear measurement provides information as to how the tear film recovers after wearing CLs, which is particularly relevant since discomfort symptoms can persist even after lens removal, especially in cases of chronic discomfort. Post‐lens wear measurements can help clarify any residual effects on the ocular surface.

One limitation of the current study was not incorporating participants with healthy eyes and others with dry eyes for comparison with CL wearers. Future research should include these groups to enhance the understanding of the effects of CLs. However, it should be noted that previous investigations have examined the repeatability of pre‐corneal LLT in both normal and dry eyes. But to our knowledge, no prior publications have assessed the repeatability of specific parameters during CL use. This study, therefore, offers a unique contribution by evaluating and comparing the repeatability of these parameters before and after the application of CLs composed of different materials. Future experiments should examine whether it is feasible to measure LLT over the CLs using the LipiView® or other devices, the reproducibility of the results and whether they are related to CL comfort.

In conclusion, the repeatability of the average, maximum and minimum pre‐corneal LLT obtained with the LipiView® device remained stable after CL wear. There was no significant correlation found between the LLT and comfort scores.

## AUTHOR CONTRIBUTIONS

**Mukesh Kumar:** Conceptualization (equal); data curation (equal); investigation (equal); methodology (equal); resources (equal); software (equal); writing – original draft (equal); writing – review and editing (equal). **Simin Masoudi:** Investigation (equal); supervision (equal); writing – review and editing (equal). **Ajay Kumar Vijay:** Conceptualization (equal); methodology (equal); supervision (equal); writing – review and editing (equal). **Thomas John Naduvilath:** Investigation (supporting); supervision (equal); writing – review and editing (supporting). **Srikanth Dumpati:** Data curation (equal); investigation (equal); writing – review and editing (supporting). **Ankit Raj:** Data curation (supporting); methodology (supporting); writing – review and editing (supporting). **Mark Willcox:** Data curation (equal); investigation (equal); methodology (equal); supervision (equal); writing – review and editing (equal).

## FUNDING INFORMATION

None of the authors have financial or proprietary interest in any material, device or methods mentioned.

## CONFLICT OF INTEREST STATEMENT

The authors report no conflicts of interest.
